# Supplementary material for: Real-World Evidence on Disease Burden and Economic Impact of Sickle Cell Disease in Italy
Source: J Clin Med. 2022 Dec 23;12(1):117. doi: 10.3390/jcm12010117 (PMC9821386; doi:10.3390/jcm12010117)
Supplement: Supplementary file 1 [file jcm-12-00117-s001.zip › jcm-2065241-supplementary.pdf]

Supplementary Tables

Table S1. International Classification of Diseases, Ninth Revision, Clinical Modification (ICD-9-CM) diagnosis codes of sickle cell disease forms

**Types of sickle cell disease form**	**ICD-9-Codes**
Sickle-cell thalassemia without crisis	282.41
Sickle-cell thalassemia with crisis	282.42
Sickle-cell disease, unspecified	282.60
Hb-SS disease without crisis	282.61
Hb-SS disease with crisis	282.62
Sickle-cell/Hb-C disease without crisis	282.63
Sickle-cell/Hb-C disease with crisis	282.64
Other sickle-cell disease without crisis	282.68
Other sickle-cell disease with crisis	282.69
Sickle-cell trait*	282.5

**not included in the analysis*

Table S2. List of SCD treatments in analysis

**Category**	**Classes/Drugs**	**Anatomical Therapeutic Chemical (ATC) codes**
SCD specific treatment	Hydroxyurea	L01XX05
SCD related drugs	Antibacterials for systemic use	J01
	Anti-inflammatory products	M01
	Analgesics	N02
SCD-complications related drugs	Drugs for functional gastrointestinal disorders	A03
	Antiemetics and antinauseants	A04
	Bile and liver therapy	A05
	vitamin D and analogues	A11CC
	Antithrombotic agents	B01
	Antianemic preparations	B03
	Albumin	B05AA01
	Cardiovascular system	C
	Drugs used in erectile dysfunction	G04BE
	Testosterone-5-alpha reductase inhibitors	G04CB
	Corticosteroids for systemic use	H02
	Antimycotics for systemic use	J02
	Immune sera and immunoglobulins	J06
	immunosuppressants	L04
	Muscle relaxants	M03
	Drugs for treatment of bone diseases	M05
	Drugs for obstructive airway diseases	R03

Corticosteroids, plain	S01BA
Anti-neovascularisation agents	S01LA
Iron chelating agents	V03AC
Drugs for treatment of hyperkalemia and hyperphosphatemia	V03AE

Table S3. International Classification of Diseases, Ninth Revision, Clinical Modification (ICD-9-CM) diagnosis codes of sickle cell related complications**.**

Systems	Types of diseases	ICD-9-Codes
**Cerebrovascular**	Stroke	430, 431, 433.x1, 434.x1, 436.x
	Transient ischemic attack	435.x
	Seizures	345.0x-345.5x, 345.7x-345.9x, 780.39
**Hepatic**	Bilirubinemia	782.4
	Gallstones	574.x
	Cholecystitis	574.00, 574.01, 574.30, 574.31, 574.60, 574.61, 575.0, 575.1x
	Biliary sludge	576.8
	Acute choledocholithiasis	574.5x
	Acute hepatic sequestration	573.8
	Acute intrahepatic cholestasis	576.8
**Renal**	Hematuria	599.7x, 791.2
	Proteinuria	791.0
	Nephrolithiasis	592.x, 594.x, 788.0, 274.11
	Polyuria	788.42
	Renal insufficiency	593.9, 585.9
	Glomerulonephritis	580.x, 582.x
**Pulmonary**	Pneumococcal pneumonia	481
	Pulmonary embolism	673.2x, 673.8x, 415.1
	Pulmonary hypertension	416.0
	Acute Chest Syndrome	517.3
	Asthma	493.0–493.9
**Spleen**	Splenic sequestration	289.52
	Hypersplenism	289.4
**Other**	Retinopathy	362.1
	Leg ulcers	707.1x
	Obstructive sleep apnea	327.23
	Aseptic (Avascular) bone necrosis	733.4
	Deep Vein Thrombosis/Thrombosis	444.x, 451-453, 671.3, 671.4, 671.9
	Hand foot syndrome (Dactylitis)	282.62, 282.64, 282.69
